# Distal leg epidermal nerve fiber density as a surrogate marker of HIV-associated sensory neuropathy risk: risk factors and change following initial antiretroviral therapy

**DOI:** 10.1007/s13365-015-0352-0

**Published:** 2015-05-22

**Authors:** Cecilia M. Shikuma, Kara Bennett, Jintanat Ananworanich, Mariana Gerschenson, Nipat Teeratakulpisarn, Tanate Jadwattanakul, Victor DeGruttola, Justin C. McArthur, Gigi Ebenezer, Nitiya Chomchey, Pairoa Praihirunkit, Piranun Hongchookiat, Pornpen Mathajittiphun, Beau Nakamoto, Peter Hauer, Praphan Phanuphak, Nittaya Phanuphak, for the SEARCH 003 protocol team

**Affiliations:** University of Hawaii, Honolulu, HI USA; South East Asia Research Collaboration with Hawaii, Bangkok, Thailand; Bennett Statistical Consulting, Inc, Ballston Lake, NY USA; US Military HIV Research Program, Walter Reed Army Institute of Research and Henry M. Jackson Foundation for the Advancement of Military Medicine, Bethesda, MD USA; The Thai Red Cross AIDS Research Center, Bangkok, Thailand; Queen Savang Vadhana Memorial Hospital, Chonburi, Thailand; Harvard School of Public Health, Boston, MA USA; Johns Hopkins School of Medicine, Baltimore, MD USA; Straub Medical Center, Honolulu, HI USA; Hawaii Center for AIDS, John A. Burns School of Medicine, University of Hawaii at Manoa, 651 Ilalo St. BSB Suite 231, Honolulu, HI 96813 USA

**Keywords:** HIV, Neuropathy, Epidermal nerve fiber density, Stavudine

## Abstract

Distal leg epidermal nerve fiber density (ENFD) is a validated predictor of HIV sensory neuropathy (SN) risk. We assessed how ENFD is impacted by initiation of first-time antiretroviral therapy (ART) in subjects free of neuropathy and how it is altered when mitochondrial toxic nucleoside medications are used as part of ART. Serial changes in proximal thigh and distal leg ENFD were examined over 72 weeks in 150 Thai subjects randomized to a regimen of stavudine (d4T) switching to zidovudine (ZDV) at 24 weeks vs ZDV vs tenofovir (TDF) for the entire duration of study, all given in combination with nevirapine. We found individual variations in ENFD change, with almost equal number of subjects who decreased or increased their distal leg ENFD over 72 weeks and no relationship to nucleoside backbone or to development of neuropathic signs or symptoms. Lower baseline distal leg ENFD and greater increases in mitochondrial oxidative phosphorylation complex I (CI) activity were associated with larger increases in distal leg ENFD over 72 weeks. Distal leg ENFD correlated with body composition parameters (body surface area, body mass index, height) as well as with blood pressure measurements. Assessed together with a companion cross-sectional study, we found that mean distal leg ENFD in all HIV+ subjects was lower than in HIV− subjects but similar among HIV+ groups whether ART-naïve or on d4T with/without neuropathy/neuropathic symptoms. The utility of ENFD as a useful predictor of small unmyelinated nerve fiber damage and neuropathy risk in HIV may be limited in certain populations.

## Background

HIV-associated sensory neuropathy (HIV-SN) is a neurologic complication of HIV characterized by bilateral lower extremity burning pain and numbness. The etiologies for HIV-SN include HIV immunovirologic factors as well as mitochondrial toxic medications such as stavudine (d4T), a medication still in use in many developing countries as part of the nucleoside reverse transcriptase inhibitor (NRTI) backbone of combination antiretroviral therapy (ART).

Distal leg epidermal nerve fiber density (ENFD) is commonly accepted as a useful predictor of small unmyelinated nerve fiber damage and of neuropathy risk in many diseases including HIV (England et al. 2009; Simpson et al. 2006; Polydefkis et al. 2002). Our study conducted serial skin punch biopsies with determination of ENFD within South East Asia Research Collaboration with Hawaii (SEARCH) 003, an open-label, randomized, clinical trial which recruited and randomized 150 HIV+ subjects in Thailand free of neuropathy prior to first-time ART to three different ART regimens differing by NRTI backbone. Specifically, a backbone of 24 weeks of stavudine (d4T) followed by switch to zidovudine (ZDV) was compared to continuous ZDV and to continuous tenofovir (TDF) for the entire 72-week duration of the study. Using ENFD as a surrogate marker of HIV-SN risk, our overall intent was to investigate the risk factors and course of HIV-SN over 72 weeks of ART.

We have previously reported on baseline immunovirologic and mitochondrial correlates of ENFD in SEARCH 003 prior to initiation of first-time ART (Shikuma et al. 2012). This present report describes the longitudinal change in ENFD seen over 72 weeks of first-time ART therapy within SEARCH 003 and the correlates of such change. We attempted to examine whether use of ART improved ENFD over 72 weeks and whether use of the neurotoxic NRTI medication d4T altered such changes in ENFD. We sought to examine the relationship of ENFD to development of SN signs/symptoms and to assess the association of various demographic, immunovirologic, and mitochondrial parameters to change in ENFD from baseline to 72 weeks. In order to identify the general ranges of ENFD that might be expected in SEARCH 003, a concurrent cross-sectional study (SEARCH 014) assessed ENFD values in healthy HIV seronegative controls and in three different cohorts of HIV+ subjects on d4T-containing ART regimens: subjects without HIV-SN, those with asymptomatic HIV-SN, and those with symptomatic HIV-SN.

## Methods

### Study design

SEARCH (http://www.searchthailand.org/) 003 was a 150-patient, 72-week, two-site clinical trial in ART-naïve subjects conducted in Thailand at the Thai Red Cross AIDS Research Centre (TRCARC) in Bangkok and at the Queen Savang Vadhana Memorial Hospital in Chonburi, Thailand (www.clinicaltrials.gov identification NCT00669487). SEARCH 003 compared, in randomized fashion, rates of anemia, lipoatrophy, and neuropathy among three ART regimens differing by NRTI backbone (Phanuphak et al. 2012). Specifically, ART-naïve Thai HIV-infected adults were randomized 1:1:1 to arm A: 24 weeks of d4T 30 mg + lamivudine (3TC) 150 mg + nevirapine (NVP) 200 mg [GPO-VIR S30^®^] twice daily followed by 48 weeks of ZDV 250 mg + 3TC 150 mg + NVP 200 mg [GPO-VIR Z250^®^] twice daily; arm B: 72 weeks of GPO-VIR Z250^®^ twice daily; and arm C: 72 weeks of TDF + emtricitabine [FTC] (Truvada^®^) once daily + NVP (Neravir^®^) 200 mg twice daily.

Clinical evaluation for HIV-SN and skin punch biopsies for distal leg and proximal thigh ENFD assessments were performed as elective procedures at baseline, week 24, and week 72 to allow an in-depth evaluation of ENFD as a surrogate marker of neuropathy risk during ART. The main ENFD findings at baseline from this study have been previously published (Shikuma et al. 2012).

Entry criteria included documented HIV infection, age >18 years, CD4 T-lymphocyte count <350 cells/mm^3^ and ART-naïve status except for females with past exposure to ART associated with pregnancy who were allowed to enroll as long as the exposure was at least 3 months prior to entry. The study utilized an entry criteria of CD4 T-lymphocyte count <350 cells/mm^3^ to be consistent with Thai national guidelines at that time for initiation of ART. Subjects were excluded for active AIDS-defining illness or other active illness, current use of immunomodulator therapy or any experimental therapy, pregnancy or breastfeeding, and presence of active malignancies. Entry criteria required subjects at entry to be free of neuropathy because of concerns about increased risk of neuropathy if randomized to the d4T-containing arm. This study was approved by the Chulalongkorn University Institutional Review Board (IRB) and the Queen Savang Vadhana Memorial Hospital IRB as primary IRBs of record and by the University of Hawaii Committee on Human Subjects as a secondary IRB. Informed consents were obtained from all subjects.

To investigate the magnitude of ENFD changes seen on ART in SEARCH 003, a concurrently running SEARCH 014 study proposed to gather cross-sectional ENFD data on 50 HIV seronegative controls and on three different groups of 25 HIV+ subjects each who were already on d4T-containing ART: subjects without neuropathy, subjects with asymptomatic neuropathy, and subjects with symptomatic neuropathy. Subjects in SEARCH 014 underwent identical evaluations as SEARCH 003 subjects including identical clinical neuropathy evaluations and distal leg and proximal thigh ENFD assessments but at a single time point. The three groups of HIV+ subjects on d4T were required to have been on d4T treatment for >6 months and to have plasma HIV RNA by PCR <50 copies/mL. HIV ELISA was utilized to confirm absence of HIV infection in HIV seronegative subjects. In order to be able to ascertain the specific effect of d4T use on ENFD, subjects were excluded from SEARCH 014 if they were on treatment with concomitant medications (other than their d4T-based antiretroviral regimen) or had conditions (e.g., compressive neuropathy, diabetes, vitamin B_12_ deficiency, excessive alcohol intake meeting substance dependence criteria by DSM-IV, hepatitis C infection) known to cause neuropathy. The presence of risk factors for neuropathy was not an exclusion in SEARCH 003. Classification into neuropathy categories utilized the same definition as that in SEARCH 003 as defined below.

### Clinical evaluations including assessment for neuropathy

Socio-demographic and medical history including relevant HIV history was obtained and a general physical examination with assessment for neuropathy performed in all SEARCH 003 and SEARCH 014 subjects. Blood was obtained for CBC, chemistries, T cell subsets, and HIV RNA as relevant. In SEARCH 003, updated medical history and physical examination including assessment for neuropathy were repeated at week 24 and 72 of study.

The evaluation for neuropathy was based on the AIDS Clinical Trials Group (ACTG)/Neurology and Neurologic AIDS Research Consortium (NARC) methodology (Simpson et al. 2006). Subjects were classified as having signs of neuropathy if they had any of the following: absent or diminished ankle reflex, diminished vibratory, pin or temperature sensation, or contact allodynia. The ACTG definition was used for peripheral neuropathy which was reduced vibration sensation in both great toes or absent or diminished ankle reflexes bilaterally relative to the knees. Subjects were classified as having symptomatic neuropathy if complaints of numbness, pain, or tingling were elicited.

Serially at entry, week 24, and 72 of study in SEARCH 003 and cross-sectionally in SEARCH 014, blood was obtained and processed on site with separation of viably preserved peripheral blood mononuclear cells (PBMCs) from plasma. PBMC was cryopreserved and shipped in batches to the University of Hawaii for various research analyses.

### ENFD assessment

**Fig. 1:**
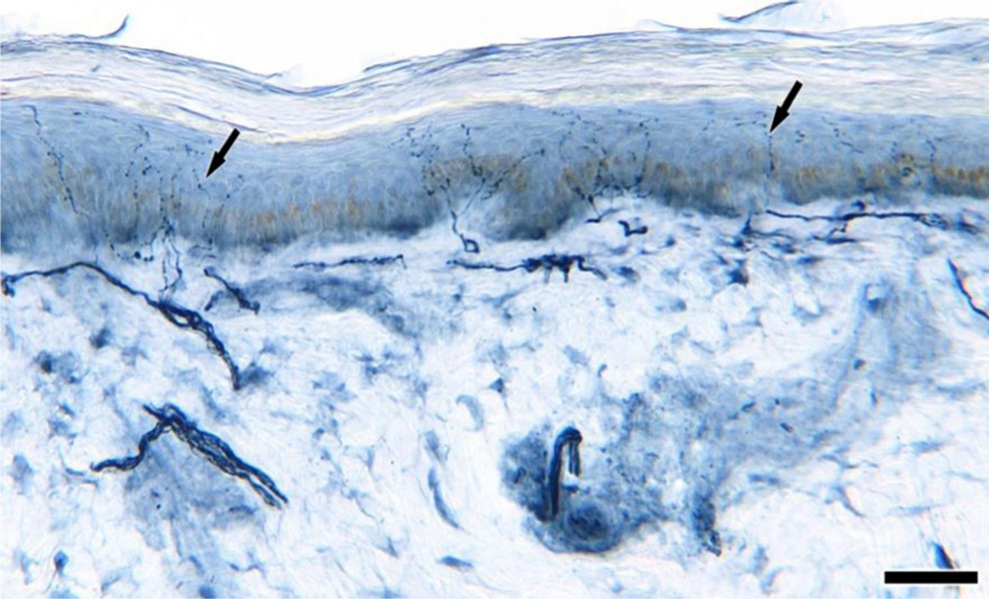
Skin biopsy section immunostained with PGP9.5 distal leg skin section from an ART-naïve subject showing numerous sensory nerve fibers (*arrows*) entering from the dermis into epidermis. *Scale bar* = 50 μm

Skin punch biopsies for ENFD were conducted longitudinally in SEARCH 003 at week 0 (entry), week 24, and week 72 and as one-time evaluations in all subjects in SEARCH 014. Skin punch biopsy samples were obtained and processed using the skin punch biopsy technique and processing recommendations of the Cutaneous Nerve Laboratory at Johns Hopkins (http://www.hopkinsmedicine.org/neurology_neurosurgery/specialty_areas/cutaneous_nerve_lab/). Briefly, following a 1 % lidocaine subcutaneous injection and utilizing sterile techniques, a 4-mm skin punch biopsy was performed on the distal leg at the level of the ankle with an additional skin punch biopsy of the upper lateral thigh. Skin specimens were processed on site and forwarded, via the University of Hawaii, to the Cutaneous Nerve Laboratory at Johns Hopkins for PGP9.5 immunostaining. To ensure acceptable specimen quality, the skin biopsies were grossly examined under a microscope for pinch and crush artifacts and such specimens were excluded from the study. Slides of 50-μm-thick immunostained sections were further confirmed for acceptable staining pattern, and the number of unmyelinated nerve fibers per millimeter length of epidermis was assessed (Fig. 1).

### Assessment of PBMC mitochondrial parameters

Viably preserved PBMCs were shipped in batch to the University of Hawaii. PBMC mitochondrial DNA (mtDNA) copies/cell were assayed by absolute quantitative real-time polymerase chain reaction (PCR) (Gerschenson et al. 2005). PBMC oxidative phosphorylation (OXPHOS) complex I (CI, NADH dehydrogenase) and complex IV (CIV, cytochrome *c* oxidase) enzyme activities were performed in duplicate by thin-layer chromatography and immunoassays (Shikuma et al. 2012; Gerschenson et al. 2009). PBMC mitochondrial-specific 8-oxo-2′-deoxyguanosine (mt 8-oxo-dG), which quantities the break frequencies in mtDNA, was assessed using a gene-specific repair assay (Shikuma et al. 2012; Brogly et al. 2011).

### Statistical methods

Hypothesis tests of changes from baseline to later time points in ENFD values were undertaken using a paired *t* test. The presence/absence of peripheral neuropathy and neuropathic signs and symptoms were compared across groups using Fisher’s exact test. Differences in quantitative variables between groups were tested using two-sample *t* test or ANOVA when comparing across more than two groups. The relationship between treatment arm and ENFD in distal leg across baseline, week 24, and week 72 was also assessed in a repeated measures ANOVA analysis. Similarly, a repeated measures ANOVA assessed the relationship between ENFDs in thigh across the three study time points. Correlations between ENFD values and other important biomarkers were assessed by Spearman correlations. Multiple regression analyses were used to assess the association between such biomarkers and the change from baseline to study week 72 in distal leg ENFD. The selection of which body composition variable (body mass index [BMI], body surface area [BSA], or height) to use was based on the adjusted *R*^2^ value.

## Results

### Longitudinal changes and differences by arms

The baseline characteristics of the SEARCH 003 subjects have previously been reported (Shikuma et al. 2012). In brief, all subjects were Thai with 56.1 % recruited from the TRCARC and 43.9 % from Queen Savang Vadhana Memorial Hospital. The median (Q1–Q3) age was 34 years (29–40), 45 % were male, 1.4 % had positive antibodies for HCV, 3.8 % had current or past use of isoniazid, and 0.7 % were diabetic as defined by entry fasting glucose >125 mg/dL. Of the 150 subjects randomized in SEARCH 003, two subjects were excluded—one for neuropathy at baseline and one became pregnant. Of the remaining 148 subjects in the analysis data set, skin punch biopsies were obtained at baseline in 133 subjects. Evaluable distal leg ENFD results were available in 120 subjects at week 24 and in 118 subjects at week 72. A total of 104 subjects had evaluable ENFD results at all time points (37 in the d4T to ZDV switch arm, 34 in the ZDV arm, and 33 in the TDF arm). The median [interquartile range] ENFD (fibers/mm) values as well as various other parameters of interest at entry, week 24, and at week 72 are shown in Table 1.

**Table 1:** SEARCH 003 patient characteristics: various parameters in subjects enrolled in SEARCH 003 at baseline, week 24, and week 72 of study

SEARCH 003 longitudinal study				*p* value^a^	
	Baseline	Week 24	Week 72	Change at week 24	Change at week 72
# Subjects with evaluable distal leg ENFD data	*n* = 133	*n* = 120	*n* = 118		
# Subjects with evaluable proximal thigh ENFD data	*n* = 133	*n* = 122	*n* = 116		
Distal leg ENFD (#/mm)	22 (20.6, 23.4)	19.6 (18.3, 21)	19.3 (17.8, 20.9)	<0.01	<0.001
Proximal thigh ENFD (#/mm)	33.8 (31.9, 35.8)	34.8 (32.9, 36.8)	35.9 (33.8, 38.1)	0.08	0.01
Body mass index (kg/m^2^)	22.1 (21.5, 22.7)	22.5 (21.9, 23)	22.5 (21.9, 23.1)	<0.01	<0.01
Height (cm)	162 (160, 163)	. (.,.)	. (.,.)		
Lean tissue (gm)	41010 (39581, 42440)	41849 (40324, 43374)	42494 (40794, 44193)	<0.01	<0.01
Limb fat (gm)	7581 (7058, 8105)	7628 (7098, 8158)	7620 (7084, 8157)	0.37	0.70
Truncal fat (gm)	5818 (5359, 6276)	5878 (5406, 6351)	6078 (5576, 6580)	0.69	0.10
Body surface area (m^2^)	1.61 (1.58, 1.64)	1.62 (1.59, 1.65)	1.62 (1.59, 1.65)	<0.01	<0.01
CD4 count (cells/mm^3^)	160.5 (145.2, 175.9)	298.8 (274.4, 323.1)	354.4 (330.7, 378.2)	<0.01	<0.01
Log10 HIV RNA (copies/mL)	4.87 (4.77, 4.98)	1.72 (1.65, 1.79)	1.68 (1.61, 1.74)	<0.01	<0.01
HIV RNA <50 copies/mL	0 % (0/0)	84 % (121/144)	93 % (132/142)	<0.01	<0.01
HOMA-IR	1.11 (0.99, 1.24)	1.38 (1.24, 1.52)	1.54 (1.38, 1.69)	<0.01	0.08
Serum lactate (mmol/dL)	0.12 (0.11, 0.13)	0.11 (0.11, 0.12)	0.11 (0.1, 0.12)	0.16	0.01
Mitochondrial DNA (copies/cell)	197.7 (184.3, 211.1)	254.8 (239.1, 270.6)	334 (312.3, 355.6)	<0.01	<0.01
OXPHOS CI enzyme activity (optical density, mg × 10^3^)	32.9 (31.2, 34.5)	32.2 (30.9, 33.6)	33 (31.5, 34.4)	0.52	0.63
OXPHOS CIV enzyme activity (optical density, mg × 10^3^)	59 (56.7, 61.4)	61.6 (59.6, 63.6)	63 (61.2, 64.9)	0.02	<0.01
Mt 8-oxo-dG (break frequencies)	0.12 (0.09, 0.14)	0.1 (0.08, 0.12)	0.04 (0.03, 0.06)		
Presence of 8-oxo-dG (if >0.1)	35 % (52/148)	34 % (49/144)	18 % (25/142)	0.79	<0.01

Parameters are expressed as median [interquartile range]. *p* values are based on the *t* test in all cases except for HOMA-IR and presence of 8-oxo-dG. For HOMA-IR, the *p* values are calculated taking the interval censoring into account using methods applied in survival analyses. For presence of 8-oxo-dG, the McNemar’s test was used

*ENFD* epidermal nerve fiber density, *HOMA-IR* homeostasis model assessment-estimated insulin resistance, *OXPHOS CI* oxidative phosphorylation complex 1 (NADH dehydrogenase), *OXPHOS CIV* oxidative phosphorylation complex IV (cytochrome c oxidase), *mt 8-oxo-dG* mitochondrial-specific 8-oxo-2′-deoxyguanosine)

^a^The null hypothesis is that there is no change (i.e., change = 0)

**Fig. 2:**
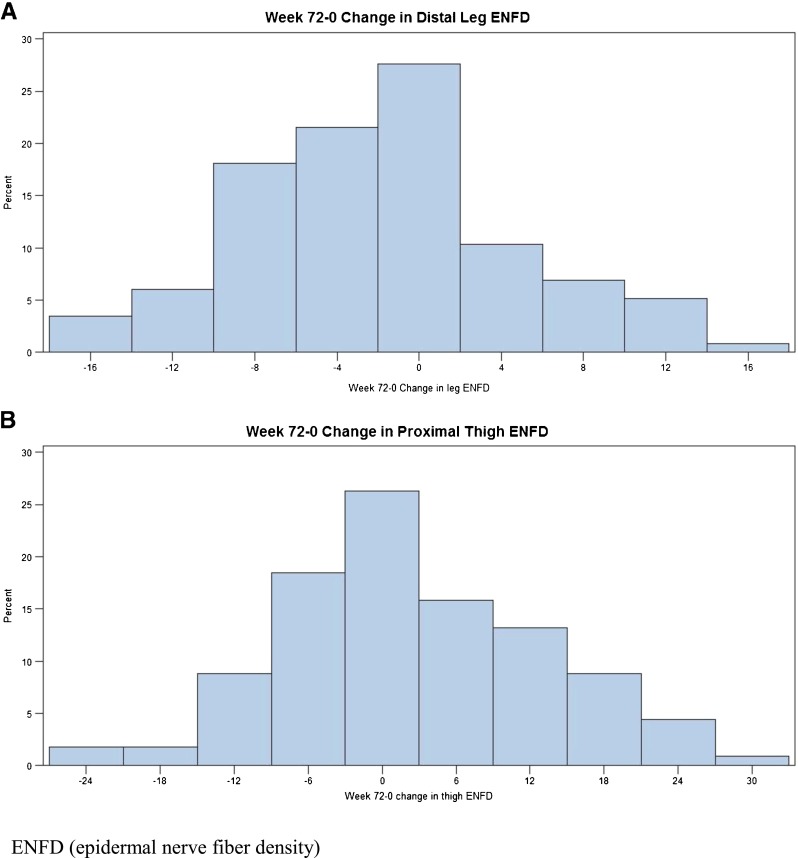
Variation in change in distal leg and proximal thigh ENFDs over 72 weeks. Histogram of percent of subjects showing various ENFD changes from baseline to 72 weeks in distal leg ENFD (**a**) and in proximal thigh ENFD (**b**) overall

**Fig. 3:**
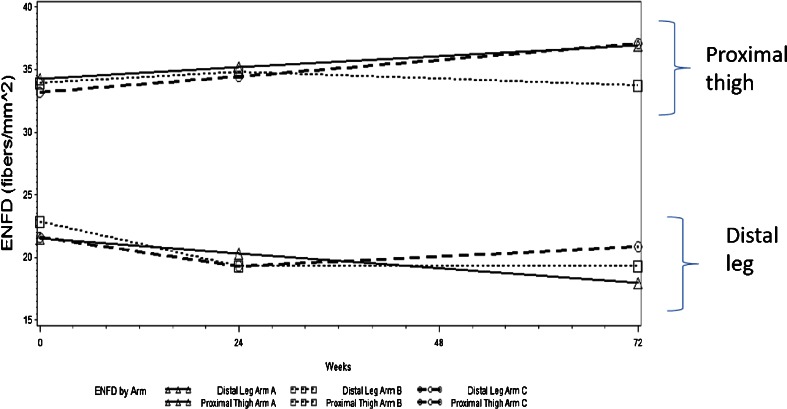
Mean distal leg and proximal thigh ENFD values over 72 weeks by arms. Distal leg and proximal thigh ENFDs were assessed at entry (week 0), week 24, and week 72

Both distal leg and proximal thigh ENFDs were comparable among the three treatment arms at entry. Overall, over the 72 weeks of the study, distal leg ENFD decreased with a median (Q1, Q3) decrease of −1.85 (−6.45, 1.85) fibers per millimeter and proximal thigh ENFD increased with median (Q1, Q3) increase of 1.90 (−4.3, 9.1) fibers per millimeter. By paired *T* test, the week 72 distal leg ENFD values were significantly decreased compared to week 0 values (*p* < 0.01), and the week 72 proximal thigh ENFDs were significantly increased compared to week 0 values (*p* = 0.01). The majority of the change occurred in the first 24 weeks [week 0- to 24-week change: distal leg ENFD *p* < 0.01; proximal thigh *p* = 0.08], with no significant differences in week 24 to 72 change for either distal leg or proximal thigh ENFD. Interestingly, however, as shown in Fig. 2, there was large variation among subjects in change in both proximal and distal ENFD irrespective of arm. Roughly, equal number of subjects in each arm increased or decreased their ENFD values. The mean ENFD values obtained by arm at the various time points are plotted in Fig. 3. Remarkably, there were no differences in ENFD values by arm at any time point (baseline, week 24, or week 72), nor were there differences in change from week 0 to 24 or change from week 0 to 72 by arm. Repeated measures ANOVA confirmed that there was no significant association between treatment arm and time point.

### Correlations with various parameters

#### ENFD correlations at baseline, week 24, and 72

Correlations between ENFD values and various biomarkers measured at the same time point were assessed at entry, week 24, and week 72. We previously reported that distal leg ENFD correlated with CD4 count at baseline (Shikuma et al. 2012), but this was no longer true at weeks 24 and 72. No associations were seen between ENFD values at the week 24 or 72 time points and lactate, glucose, homeostasis model assessment-estimated insulin resistance (HOMA-IR) values, or in PBMC CI or CIV values at the corresponding time points, with the exception of week 72 proximal thigh ENFD and week 72 lactate (*r*_s_ = −0.27, *p* < 0.01).

Significant (most with *p* < 0.01) negative correlations, largely consistent over all three time points, were seen between both distal leg and proximal thigh ENFD values and body composition values for height, BMI, and for BSA at their corresponding time points. In univariate linear regression models for the same time points, BSA gave the highest association based on adjusted *r*^2^ ranging from 0.06 to 0.11.

Similarly, negative correlations, consistent over all three time points, were seen between distal leg ENFD and both systolic and diastolic BP (*r*_s_ of approximately −0.3, *p* < 0.01). The relationship of blood pressure with proximal thigh ENFD was less consistent with significant (*p* < 0.05) negative correlations seen with corresponding systolic blood pressure at baseline and with corresponding diastolic blood pressure at 72 weeks only.

#### Correlations with change in ENFD

Week 0 to 24 change in distal leg ENFD correlated with both baseline distal leg ENFD (*r*_s_ = −0.53, *p* < 0.01) and baseline proximal thigh ENFD (*r*_s_ = −0.26, *p* < 0.01). In addition, week 0 to 24 change in distal leg ENFD correlated negatively with baseline CD4 (*r*_s_ = −0.37, *p* < 0.01) and positively with baseline mtDNA (*r*_s_ = 0.24, *p* < 0.01). Week 0 to 24 change in proximal thigh ENFD correlated with baseline proximal thigh ENFD (*r*_s_ = −0.33, *p* < 0.01), baseline distal leg (*r*_s_ = −0.30, *p* < 0.01), and baseline mtDNA (*r*_s_ = 0.18, *p* = 0.04).

Week 0 to 72 change in distal leg ENFD correlated with baseline distal leg ENFD (*r*_s_ = −0.38, *p* < 0.01), baseline proximal thigh ENFD (*r*_s_ = −0.20, *p* = 0.04), baseline CD4 (*r*_s_ = −0.33, *p* < 0.01), week 0 to 72 change in BSA (*r*_s_ = 0.29, *p* < 0.01), week 0 to 72 change in CI activity (*r*_s_ = 0.24, *p* = 0.01), and baseline mtDNA (*r*_s_ = 0.18, *p* = 0.05). Week 0 to 72 change in proximal thigh ENFD correlated with baseline proximal thigh ENFD (*r*_s_ = −0.35, *p* < 0.01) and baseline mtDNA (*r*_s_ = 0.20, *p* = 0.03).

Using multivariate linear regression, we further assessed the factors associated with change in distal leg ENFD over 72 weeks. As shown in Table 2, change over 72 weeks in distal leg ENFD was strongly associated with baseline distal leg ENFD (*p* < 0.01) and more weakly with change in PBMC CI levels (*p* = 0.05) and baseline CD4 count (*p* = 0.07) in a model also adjusted for treatment arm and change in BSA.

**Table 2:** Multiple regression model of predictors of change over 72 weeks in distal leg epidermal nerve fiber density in SEARCH 003

Variable	Estimate (95 % CI)	*p* value
Intercept	5.21 (1.28, 9.13)	<0.01
Tx arm A (ref = tx arm C)	−1.88 (−4.6, 0.85)	0.17
Tx arm B (ref = tx arm C)	−0.89 (−3.69, 1.9)	0.53
Baseline ENFD (distal leg)	−0.22 (−0.36, −0.08)	<0.01
Baseline CD4 (cells/mm^3^)	−0.01 (−0.03, 0)	0.07
Week 72–week 0 body surface area	15.76 (−7.38, 38.91)	0.18
PBMC: change C1 activity week 72–week 0	0.12 (0, 0.25)	0.05

Tx (treatment). Arm A: stavudine for 24 weeks followed by 48 weeks of zidovudine. Arm B: zidovudine for entire 72 weeks of study. Arm C: tenofovir for entire 72 weeks of study

*ENFD* epidermal nerve fiber density, *CI* oxidative phosphorylation complex I (NADH dehydrogenase) enzyme activity, *PBMC* peripheral blood mononuclear cells

### Relationship of ENFD to development of neuropathic signs/symptoms and neuropathy on study

At entry, as specified by the protocol, no subject had neuropathy although two subjects (one randomized to the d4T to ZDV switch arm and one to the TDF arm) had neuropathic signs at entry. The development of neuropathic signs and neuropathy in SEARCH 003 has been reported previously (Phanuphak et al. 2012). Briefly, at week 24, using intent to treat analysis, 5 of 51 (9.8 %) subjects in the d4T to ZDV switch arm, 10 of 49 (20.4 %) subjects in the ZDV arm, and 1 of 47 (2.1 %) subject in the TDF arm developed neuropathic signs and symptoms; these proportions differed significantly across treatment arms (*p* = 0.01). At 72 weeks, the number of subjects with neuropathic signs/symptoms in the d4T to ZDV switch arm was 5/51 (9.8 %) subjects, in the ZDV arm 12/49 (24.5 %) subjects, and in the TDF arm 5/47 (10.6 %) subjects; these proportions did not differ significantly (*p* = 0.09). The rates of neuropathy were 7.8, 10.2, and 0 % at week 24 and 9.8, 16.3, and 6.4 % at week 72, respectively, for the d4T to ZDV switch arm, the ZDV arm, and TDF arm. These proportions did not differ significantly across treatment arms (*p* = 0.07 for week 24 and *p* = 0.29 for week 72).

There were no differences in week 0 (entry) or week 24 distal leg or proximal thigh ENFD among those who did or did not develop neuropathic signs/symptoms at week 24, nor were there differences in week 0 to 24 change in distal leg or proximal thigh ENFD. Similarly, there were no differences by neuropathic signs/symptoms at week 72 by week 0, 24, or 72 distal leg or proximal thigh ENFD or in distal leg or proximal thigh ENFD change from week 0 to 24, week 24 to 72, or week 0 to 72. Analyses identical to above were performed comparing those with and those without peripheral neuropathy, and no significant differences were detected.

### SEARCH 003 ENFD comparison with SEARCH 014 controls

The SEARCH 014 patient characteristics are shown in Table 3. The HIV seronegative group was slightly younger than the HIV+ groups. There were some gender differences with fewer males in the HIV+ symptomatic neuropathy group. By BMI, the groups were quite similar. In terms of HIV immunovirologic parameters, the CD4 count and % with undetectable HIV RNA were similar within the HIV+ groups.

**Table 3:** SEARCH 014 patient characteristics: various parameters of subjects enrolled in SEARCH 014

SEARCH 014 cross-sectional study					*p* value^a^
	HIV negative	HIV+	HIV+	HIV+	
	No neuropathy	No neuropathy	Asymptomatic neuropathy	Symptomatic neuropathy	
*N* (# subjects)	*n* = 50	*n* = 25	*n* = 6	*n* = 16	–
Age (years)	33.3 (31.9, 34.6)	37.2 (35.2, 39.3)	38.3 (31, 45.6)	40.3 (36.6, 43.9)	<0.01
Gender (% male)	22 (44 %)	17 (68 %)	4 (66.7 %)	4 (25 %)	0.03
Height (cm)	162.3 (160.5, 164.1)	164.5 (161.2, 167.8)	166.3 (157, 175.6)	158.1 (153.2, 163)	0.03
Body mass index (kg/m^2^)	22.12 (21.45, 22.8)	21.42 (20.13, 22.71)	21.19 (18.8, 23.58)	22.4 (21.04, 23.75)	0.53
Body surface area (m^2^)	1.62 (1.59, 1.64)	1.62 (1.56, 1.68)	1.65 (1.48, 1.81)	1.57 (1.48, 1.66)	0.43
CD4 count (cells/mm^3^)	–	410.4 (331.9, 489)	444.2 (237.2, 651.2)	475.1 (362.5, 587.8)	0.60
% Undetectable HIV RNA (<50 copies/mL)	–	24 (96 %)	6 (100 %)	15 (93.8 %)	1.00
Distal leg ENFD (#/mm)	30.1 (27.1, 33.1)	21.1 (17.8, 24.4)	19.2 (13.9, 24.6)	22.3 (17.8, 26.9)	<0.01
Proximal thigh ENFD (#/mm)	46.9 (43.3, 50.4)	35.8 (29.9, 41.6)	49.7 (40.7, 58.8)	40.1 (32.1, 48.1)	<0.01

Parameters are expressed as mean (95 % CI)

^a^ *p* values based on ANOVA test with the exception of gender and undetectable HIV RNA which were based on Fisher’s exact test

Within the SEARCH 014 groups, distal leg ENFDs in all three groups of HIV+ subjects on d4T, irrespective of their neuropathy status, were significantly lower compared to HIV seronegative controls (all *p* < 0.01). There were no differences in distal leg ENFD, however, among the three groups of HIV+ subjects on d4T.

SEARCH 003 distal leg and proximal thigh values at all time points were lower compared to SEARCH 014 HIV seronegative subjects (*p* < 0.01). There were no differences between mean distal leg values at any SEARCH 003 time point and mean distal leg values of any of the SEARCH 014 d4T groups. For proximal thigh, the values at each of the SEARCH 003 time points were lower than the proximal thigh value for the SEARCH 014 HIV+ subjects on d4T with asymptomatic neuropathy (all *p* < 0.01).

## Discussion

Our study found large individual variation in change in ENFD with first-time ART initiation which was not accounted for by use of any specific NRTI regimen. Greater increases in distal leg ENFD over 72 weeks were associated with lower baseline distal leg ENFD, a trend toward lower baseline CD4 count, and greater week 0 to 72 increase in PBMC CI levels. Our study found that various body composition measurements (height, BMI, BSA) and blood pressure measurements were associated with ENFD values. No difference in ENFD values were found by neuropathic signs or symptoms, and values in subjects on first-time ART, most without neuropathy, were no different to values obtained cross-sectionally in subjects on d4T with neuropathy.

Our study suggests that ENFD as a useful predictor of small unmyelinated nerve fiber damage and of neuropathy risk in HIV may need to be reexamined in certain populations, such as in the HIV+ Thai population utilized in this study. While ENFD values were significantly lower in all HIV+ subjects (whether ART-naïve or at week 24 or week 72 post-initiation of ART in SEARCH 003 or on d4T-based therapy in SEARCH 014) than in HIV seronegative subjects, ENFD values were similar among all HIV+ groups and did not distinguish individuals with or at risk of developing neuropathy.

Despite differences in definition and methodology, rates of HIV SN in ranges as high as 50–60 % have been reported in the literature particularly with use of d4T as part of the ART regimens (Schutz and Robinson-Papp 2013). Very few subjects in our study developed neuropathic signs or symptoms on this study even in the arm utilizing d4T. This low percentage may have been due to several factors. The duration of d4T use in our study was a short 24 weeks, and a lower dose of 30 mg daily was used rather than the older 40 mg dose, a factor known to minimize risk of neuropathy (Maskew et al. 2012; Pahuja et al. 2012). We have previously reported that our Thai seronegative subjects have higher ENFD values compared to published US control ENFD values (Shikuma et al. 2013) which may have provided our Thai subjects with a higher “ENFD reserve.” We as well as others have reported the impact of underlying genetic factors on neuropathic risk (Hulgan et al. 2014; Kampira et al. 2013; Wadley et al. 2013; Domingo et al. 2013), and it is possible that such influences as well as possible differences in virologic factors may modulate development of neuropathy in different HIV-infected populations. Our study was also unusual in the development of more neuropathic signs and symptoms in the ZDV arm (Phanuphak et al. 2012), a factor that remains puzzling.

The large variation in change in ENFD values over 72 weeks in SEARCH 003, both in the distal leg and in the proximal thigh, was unexpected and suggests that parameters other than NRTI selection were more important factors of change in this study. We did find that improvement in distal leg ENFD was greater in subjects who were initiating ART with lower ENFD values and perhaps in those with lower CD4 counts who had more to gain from ART and also in subjects with greater capacity to improve energy metabolism (i.e., able to improve mitochondrial function as reflected by an increase in CI). These factors, however, only explained approximately 20 % of the variance. Similar to other noninfectious complications associated with HIV, we speculate that HIV-associated inflammatory tendencies not fully suppressed by ART and not effectively assessed by parameters examined in this study may explain part of the variance.

The relationship between height and distal ENFD has been previously reported (Cherry et al. 2009). It has been hypothesized that the higher energy demands of taller individuals resulting from a need to actively transport essential compounds down their longer processes from the dorsal root ganglia may mean that they are particularly vulnerable to any insult to these sensory nerves. We add to this information that while height showed correlations with ENFD in our study as well, composite scores of BMI and BSA, both of which utilize calculations based on weight and height, also correlate. Of the body composition measurements evaluated, we utilized BSA in the multiple regression model because it gave the most robust association with distal leg ENFD, accounting for 6 to 11 % of the variance. However, use of height or BMI also gave similar results. It can be hypothesized that the contribution of weight may be mediated via increases in weight-associated pro-inflammatory tendencies and/or other factors related to weight-induced changes in glucose metabolism. Interestingly, a possible protective effect for HIV SN with noninsulin glucose-lowering drugs has been reported (Evans et al. 2012). In the case of BSA, it can be hypothesized that this composite value may explain more of the variance than height simply because of the “stretching” of the skin leading to less nerve fiber endings per millimeter of skin. The negative relationship between blood pressure measurements and distal leg ENFD is unlikely to be the result of impaired glucose metabolism, as there was no association with HOMA-IR but may be related to concomitant dysregulating influences on autonomic nerves traveling along with the small unmyelinated nerve fibers responsible for HIV-SN.

Our study calls into some question how closely ENFD values can reflect the presence of HIV SN or predict risk in certain populations as demonstrated by the lack of a significant difference in ENFD in SEARCH 014 Thai subjects on d4T with or without neuropathy or in SEARCH 003 Thai subjects who did or did not have incident neuropathic signs or symptoms. While it has traditionally been believed that proximal thigh ENFD is relatively stable and could be used as a control for changes in distal leg ENFD, proximal thigh ENFD values were somewhat more variable in our HIV+ groups than distal leg ENFD values. Examining the data as a ratio of distal leg ENFD/proximal thigh ENFD did not help to distinguish subjects with or without neuropathy or with or without neuropathic signs or symptoms (data not shown).

This study has several limitations including the short 72-week duration of the study, the low number of subjects who developed neuropathic signs and symptoms, and its limited applicability to ENFD changes only in the Thai population. The strengths of the study are the careful quality-controlled clinical evaluation for neuropathy and the laboratory evaluation for ENFD done in a laboratory familiar with the assessment of ENFD.

In summary, our study found large individual variations in ENFD change following initial ART which was not related to nucleoside (tide) backbone. Poorer clinical status as reflected by lower distal leg ENFD and possibly lower CD4 count as well as greater ability to increase mitochondrial energetics was associated with greater increase in distal leg ENFD over 72 weeks. Body composition measurements of BSA, BMI, and height as well as blood pressure measurements correlated with ENFD. ENFD measurements did not distinguish individuals with or without neuropathy or with or without new onset of neuropathic signs/symptoms. The value of ENFD as a surrogate marker for HIV-associated SN may need to be reevaluated in certain populations.
